# A Literature Review on the Progression of Agile Manufacturing Paradigm and Its Scope of Application in Pump Industry

**DOI:** 10.1155/2015/297850

**Published:** 2015-05-12

**Authors:** V. M. M. Thilak, S. R. Devadasan, N. M. Sivaram

**Affiliations:** ^1^Department of Production Engineering, PSG College of Technology, Coimbatore, Tamil Nadu 641004, India; ^2^Department of Mechanical Engineering, National Institute of Technology, Tiruchirappalli, Tamil Nadu 620015, India

## Abstract

During the recent years, the manufacturing world has been witnessing the application of agile manufacturing paradigm. The literature review reported in this paper was carried out to study this progression. This literature review was carried out in two phases. In the first phase, the literature was reviewed to trace the origin of agile manufacturing paradigm and identify its enablers. Further, during this phase, the applications of agile manufacturing reported in literature arena were reviewed. It was also discernable that certain research works have been initiated to apply agile manufacturing paradigm in pump industry. During the second phase, the researches reported on applying agile manufacturing in pump industry were reviewed. At the end of this review, it was found that so far the implementation of agile manufacturing in pump industry has been examined by the researchers by considering only certain components of pumps. In fact, the holistic implementation of agile manufacturing in the pump industry is yet to be examined by the researchers. In the context of drawing this inference, this paper has been concluded by stating that high scope exists in examining the infusing of agility characteristics in designing and manufacturing of pumps.

## 1. Introduction

The field of manufacturing emerged in the world during ancient days when humankind began to shape the naturally available components to fulfill specific needs. Particularly, the humankind that lived during ancient days would have sharpened the stones for killing animals. Thus, the manufacturing field has ancient day roots. On further development, the manufacturing field expanded to produce little more sophisticated products like utensils and furniture. In order to manufacture these little more sophisticated products, the humankind began to adopt manufacturing paradigms. The first paradigm that the manufacturing world adopted was craft production. Craft production paradigm enabled the humankind to produce products in low volume by employing few people. After the industrial revolution, the manufacturing field witnessed the emergence of mass production paradigm. Mass production paradigm enabled the humankind to produce products in large volume by employing many people. Mass production paradigm dominated the manufacturing scenario till the 1960s. Thereafter, the manufacturing world began to witness competition. In order to face this competition, the companies started adopting lean manufacturing paradigm which focused on eliminating wastes which occurred during the mass production. From the 1980s, the world began to experience the intensification of competition. In order to face this kind of intensified competition, the world witnessed the emergence of agile manufacturing paradigm. Agile manufacturing paradigm focuses on manufacturing products based on the dynamic demands of the customers. Furthermore agile manufacturing paradigm enables an organization to supply products according to the choice and specifications of the customer. This capability is addressed by their researchers under the term “mass customization.” Today, the companies which have been able to mass-customize the products produced by them through the installation of agile manufacturing paradigm have been able to thrive in the market. Though agile manufacturing has been adding certain industries to face the competition, its application in many other industries is not significantly reported. This situation warrants the need to not only review the literature for studying the state-of-the-art researches on agile manufacturing but also explore its exploitation in an industry in which it is yet to find in-depth application. In this regard, pump manufacturing industry was chosen as the candidate industry for studying the application feasibility of agile manufacturing paradigm. The literature review reported in this paper was carried out in two phases. In the first phase, the state-of-the-art researches on agile manufacturing were studied. In the second phase, the scope applying agile manufacturing concepts in pump industry was studied. The details of carrying out the activities under these two phases are presented in the subsequent sections of this paper.

## 2. Literature Review Methodology

The literature review being reported here was begun by gathering papers reporting researches on agile manufacturing. Then, the meaning, definition, and emanation of agile manufacturing reported in these papers were studied. After reviewing this information, the enablers of agile manufacturing were identified. Subsequently, the different industries where agile manufacturing has been successfully applied were identified and studied. At this stage, the scope of the literature survey being reported here was restricted to studying the research activities on applying agile manufacturing in the pump industry. Subsequently, a search was made in the literature arena to identify the paper reporting research activities in this direction. This search resulted in the identification of three papers reporting the researches on applying agile manufacturing in the pump industry. These papers were studied in detail. The details of these activities are briefly reported in the following sections of this paper.

## 3. History, Origin, and Meaning of Agile Manufacturing

The world began to experience globalization from the 1990s. The globalization resulted in the entry of products produced by different countries into the local markets. This scenario fueled the global competition between companies situated in various parts of the world [1]. This global competition enabled the customers to demand products with innovative features at low price and high degree of quality [2–6]. In order to meet the dynamic demands of the customers, few companies in the world acquired agile capabilities to manufacture innovative products within a short period of time to withstand the global competition [7]. A very apt example to be cited to support this claim is the capabilities being demonstrated in contemporary days by the mobile phone manufacturing companies. The modern mobile phone manufacturing companies have been manufacturing and selling several models of mobile phones with amazing innovative features.

In the early period, mobile phone manufacturers produced large sized mobile phones with the provision for telephoning and utilizing other few facilities like calculator and calendar. Today, those manufacturers have brought out mobile phones under different names like tablet and iPad, which are incorporated with facilities like high resolution camera and high speed internet technology. Thus, the mobile phone manufacturers are capable of quickly responding to the dynamic demands of the customers. This is due to the fact that mobile phone manufacturing companies are incorporated with a type of production paradigm that is agile in nature. In the year 1990, a group of researchers coined the term “agile manufacturing” after seeing this kind of developments [8]. Then, researches in the direction of achieving agile manufacturing were pursued by forming agile forum at Iacocca Institute in Lehigh University, USA.

### 3.1. Definitions and Meaning of Agile Manufacturing

The formation of agile forum at Iacocca Institute in Lehigh University resulted in a considerable number of researches in effecting agile manufacturing in different companies. Initially, these researchers began to work on discovering the meaning of agile manufacturing. The common meaning recognized by researchers is that agile manufacturing is a manufacturing paradigm that enables the companies to respond to the dynamic demands of the customers quickly [9]. In the recent times, researches on the procedures to implement agile manufacturing in different manufacturing industries have been developed by the researchers. These efforts have not been successful in all the cases due to certain limitations. The high investments for implementation, rigid nature of the product, and lack of multiskilled employees are few obstacles of successfully implementing agile manufacturing in industries. Thus, smart manufacturing industry like mobile phone manufacturing industry flourishes by either implicitly or explicitly adopting agile manufacturing paradigm, whereas many traditional industries are still struggling to flourish in today's competitive world. However, some manufacturing industries which are yet to implement agile manufacturing have spontaneously imbibed agile manufacturing principles. For instance, the automobile sector has been imbibing agile manufacturing principles spontaneously, as different models of cars are manufactured in this sector to fulfill the different tastes and requirements of customers. Thus, the journey of agile manufacturing which began at Iacocca Institute at Lehigh University advances today through different manufacturing sectors.

After the formation of agile forum at Iacocca Institute at Lehigh University, USA, the researchers made efforts to define agile manufacturing. These efforts of the researchers resulted in several definitions of agile manufacturing in the literature arena. Few authors like Gunasekaran and Yusuf [10] and Jin-Hai et al. [11] have summarized these definitions. A study of these definitions would indicate that the researchers viewed agile manufacturing paradigm as the post-mass production concept which focuses on meeting the global competition by quickly responding to the dynamic demands of the customers [12]. Comprehension of these definitions indicates that agile manufacturing enables a company to be flexible enough to quickly respond to the dynamic demands of the customers and manufacture products with many varieties and innovative features [13]. The manufacturing system reconfigurability and flexibility to produce varieties of products are the critical characteristics of agile manufacturing. In other words, agile manufacturing aids the company to manufacture different varieties of products based on the dynamic changes in customer needs in a shorter period of time.

## 4. Enablers of Agile Manufacturing

After developing the definitions of agile manufacturing, researchers began to carry out studies for determining the enablers that would aid the companies with implementing agile manufacturing principles. These researches have been repeatedly reported in literature arena. The agility enablers pinpointed by the authors in these research papers are enumerated in Table 1.

An overview of the contents of Table 1 would indicate that RPT and virtual manufacturing are found to be often mentioned as enablers of agile manufacturing. RPT aids the rapid production of prototypes which enable the companies to reconfigure the products to suit the customers' preferences before the product is actually manufactured in the shop floor. Furthermore, virtual manufacturing enables the viewing of manufacturing procedure in the computer screens before the product is actually manufactured. This capability of virtual manufacturing enables the reconfiguration of manufacturing facilities to suit the manufacturing of the products according to the customers' dynamic demands [14]. An interesting observation made at this juncture was that, during the recent years, researchers have been appraising the power of cloud manufacturing model in enhancing the virtual manufacturing and reconfigurable capabilities of organizations for acquiring agility characteristics. Cloud manufacturing model has the potential to aid the organizations to gather necessary knowledge at low price to become agile companies through the utilization of Information Technology resources [15–19]. Next to RPT and virtual manufacturing, CAD is frequently referred by the researchers as agile enabler. Critical thinking would specify that, without the aid of CAD, the production of prototypes rapidly using RPT and reconfiguring the manufacturing facilities by using virtual manufacturing is not possible. Thus, CAD aids the reconfiguration of products and manufacturing facilities. Hence, the foundational enabler of agile manufacturing is the application of CAD in the case of manufacturing products.

## 5. Industrial Applications of Agile Manufacturing

**Figure 1:**
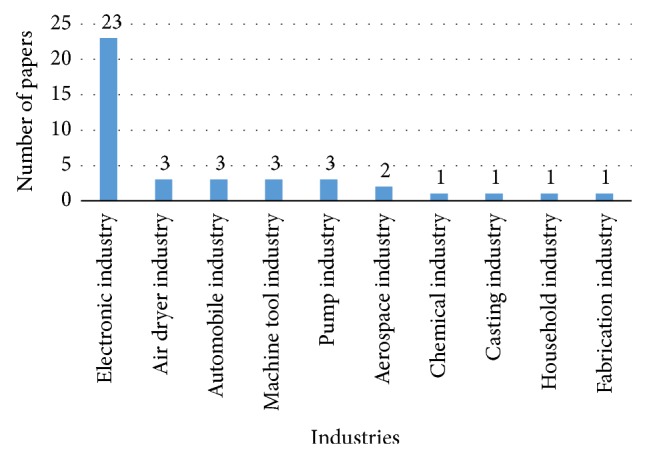
Bar chart of industrial applications of agile manufacturing.

Several researchers have endeavored to implement agile manufacturing paradigm in different industries right from its conceptualization. This resulted in appearance of many research papers reporting the experiences of implementing agile manufacturing in various industrial sectors in the literature arena. During the literature review being reported here, a total of 48 research papers on industrial application of agile manufacturing were found. The nature of researches is highlighted in Table 2. The statistical aspects of the paper reviewed are presented in the bar chart shown in Figure 1. As shown, the number of papers reporting the application of agile manufacturing in electronics industry is maximum compared to the number of papers reporting researches on agile manufacturing in other industries. Since various types of researches on agile manufacturing in electronic industry are reported, their nature is described in the next subsection.

### 5.1. Application of Agile Manufacturing in Electronics Industry

The highlights of the researches reported on implementing agile manufacturing researches in electronic industries are a little more elaborated in Table 3. As shown, the researchers have chosen four products, namely, electronic switch, printed circuit board, computer, and semiconductor while pursuing researches on implementing agile manufacturing. Besides, electronic components were also chosen as the candidate product while pursuing such researches. A careful study of the contents presented in Table 3 would indicate that these researchers have applied several technologies like CAD, CAM and RPT, and e-CRM. Thus, the trace of implementing agile manufacturing in electronic industry has been faster than it is done in the case of other industries. This trend indicates the need to identify the ways of increasing the pace of agile manufacturing in other industries too. As mentioned earlier, in order to initiate research in this direction, the application of agile manufacturing in pump industry is explored in the next subsection.

### 5.2. Application of Agile Manufacturing in Pump Industry

A few authors have reported researches on implementing agile manufacturing in pump industry. These researches are briefly described here. Vinodh et al. [20] have carried out a research to analyze the impact of using CAD and RPT in achieving agility in pump industry. These authors chose the impeller of the centrifugal pump as the candidate product and created its new designs using CAD. Then, these new designs were subjected to flow analysis using two software packages, namely, Gambit and Fluent. These authors chose one new design after conducting the analysis and a prototype of that design was created using a rapid prototyping machine. These steps indicated that the new innovative products can be brought out in the industry by using CAD, CAE, and RPT. These authors carried out an activity based costing methodology to determine the costing of manufacturing the impeller. This research proved that CAD and RPT can be used to infuse agility in pump industry.

A case study on assessing the agility level of a traditional pump industry was carried out by Vinodh et al. [21] using a 30-agile-manufacturing-criterion quantification tool. These authors assessed the agility index of the company by collecting the responses from the executives of the company. The score obtained by using the agile manufacturing criteria quantification tool was 529 out of 1000. Since this score is above 500, these authors interpreted that agile manufacturing could be infused in this traditional pump manufacturing industry. Then, the gap analysis was carried out based on the obtained scores. Based on the results obtained, these authors proposed activities to infuse agile characteristics in the pump manufacturing industry.

Devadasan et al. [22] have reported a research on employing Taguchi's offline model in a traditional pump manufacturing company. The literature survey carried out by these authors indicated that the experiments including Taguchi's offline model have been conducted by considering only quantitative features. But in an agile manufacturing environment, the qualitative features should also be considered for carrying out these experiments. Since the cost of conducting actual experiments involves large amount of time and money, these authors preferred conducting of interviews. The interviews conducted were based on Delphi approach. These authors carried out a case study in a submersible pump manufacturing company to assess the success rate of the experiments. In the first phase of the experiment, these authors identified the factors which would aid in infusing agility while designing a new model of pump. Then, using Taguchi's orthogonal array based approach, the experiments containing quantitative and qualitative factors and levels were designed. These authors showed these experiments to two top executives of that company and gathered their feedbacks. In the second phase of the case study, the possibilities of infusing 20 agile criteria were analyzed and it was found that 11 out of 20 criteria 7 are feasible to infuse agility in this company. Since the cost of conducting experiments is more, instead of conducting experiments, these authors interviewed two executives. The response of the executives indicated that there are possibilities of achieving agility in a traditional pump manufacturing industry.

In total, the researches reported in the above papers specified that several agile criteria need to be strengthened in the pump manufacturing industry in several stages. The results of the literature review indicated that the assessment tool which is designed based on agility criteria is useful in determining the gaps to be filled in pump manufacturing industry to adopt agile manufacturing.

## 6. Conclusion

The literature review reported in this paper was carried out with the motivation behind following the progression of agile manufacturing in the manufacturing world. This literature review was first started by evaluating the looks into investigated agile manufacturing in the literature arena. It was discovered that, to start with, researchers tried exertions to pinpoint the significance and meaning of the term agile manufacturing and its related terms. In the wake of this intersection, the researchers worked towards figuring out the influences of agile manufacturing. During the current phase, the researchers are endeavoring to infuse agility in the manufacturing of traditional products. Throughout the conduct of the literature review reported in this paper, it was observed that just a couple of researchers have worked toward implanting agility in the manufacturing of pumps. Actually, in the researches reported in these papers, just an advance has been made to infuse agile characteristics in the manufacturing of certain components of pumps. Similarly, an exhaustive methodology of infusing agile characteristics while manufacturing pumps has not been addressed in those papers. An interesting observation made while conducting this literature review was that many researchers have attempted to infuse agility characteristics in electronic industry. It is presumed that this has happened so, as the electronic products generally hesitate for digitization. The digitization enables the development of new products in an agile manner through virtual analysis using computers. However, in the case of other products, particularly those developed before digitization technologies emerged in the world, the practice of achieving agility through digitization has been a challenging task. Hence, the modern researchers have begun to make attempts for digitizing these kinds of traditional products. Among these products, the pump fell within the scope of the literature review reported in this paper. Hence, during the conduct of the literature review reported in this paper, the progression of agile manufacturing happening toward infusing agile manufacturing in pump industry was traced. At the end of tracing this progression of agile manufacturing, it is pointed out that there are possibilities of carrying out investigations on infusing agility characteristics while designing and manufacturing of pumps. This sort of research attempts will help the traditional pump manufacturers to generate profit like the way the cell phone manufactures have garnered.

## Figures and Tables

**Table 1:** Agility enablers reported in literature arena.

Paper number	Papers reporting agility enablers	Number of agility enablers identified	Agility enablers
1	Vinodh et al. [23]	2	Computer-Aided Design (CAD), Rapid Prototyping Technology (RPT).

2	Yusuf et al. [24]	4	Core competence management, Virtual Enterprise (VE) formation, capability for reconfiguration, and knowledge driven enterprise.

3	Narasimhan et al. [25]	4	Partnership arrangement, close relationships with suppliers, JIT production, and advanced technologies.

4	Vinodh et al. [26]	5	Organizational structure enabler, manufacturing management enabler, employee agility enabler, technology enabler, and manufacturing strategy enabler.

5	Gunasekaran [27] Bottani [28] Gunasekaran and Yusuf [10]	7	VE formation tools/metrics, physically distributed teams, and manufacturing, rapid partnership formation tools/metrics, Concurrent Engineering (CE), integrated product/production, business information system, rapid prototyping tools, electronic commerce (EC).

6	Vinodh and Kuttalingam [9]	9	CAD, Computer Aided Manufacturing (CAM), Computer Numerical Control (CNC), computer-integrated manufacturing (CIM), Rapid Prototyping (RP), Rapid Tooling (RT), Reverse Engineering (RE), VE, Information Technology (IT).

7	Dowlatshahi and Cao [29]	10	Core competencies, VE, RPT, CE, multiskilled and flexible people, continuous improvement, team working, change and risk management, IT, empowering.

8	Sreenivasa and Devadasan [30]	25	Artificial Intelligence (AI), internet, inter- and intraenterprise activities, Standard for Exchange of Products (STEP), CE, virtual manufacturing, component based distributed shop floor control system, tabu-enhanced genetic algorithm approach, customization, RPT, IT, AI, web technology, innovative total quality function deployment (ITQFD), management responsibility, manufacturing management, employee status, technology and manufacturing strategy, RPT, Computer-Aided Design of Experiments (CADOE), activity based costing (ABC), CAD/CAM, CAD, and RPT.

9	Eshlaghy et al. [31]	25	VE, EC, RPT, improvement, multiskilled and flexible people, team working, CE, change and risk management, integrated information system, continuous improvement, flexible infrastructure, supply chain, improved manufacturing technology, core competence, capability for reconfiguration, knowledge management, innovation, agile strategy, agility process, reward system, culture, reengineering, leadership, collaborative relationships, uncertainty and essential changes, people leverage, and IT.

10	Vázquez-Bustelo et al. [32]	53	Top management support, employee involvement and empowerment, team working, job rotation (multifunctional workforce), training and education, knowledge workers, decentralized decision making, entrepreneurial firm culture, reward schemes to encourage innovation and based on both financial and nonfinancial measures, Enterprise Resource Planning (ERP), Materials Requirement Planning (MRP), Robotics, automated guided vehicle (AGV), automated storage and retrieval systems, CNC machines, CAD/CAM, RPT tools, intranet internet and world wide web, Electronic Data Interchange (EDI), EC, visual inspection, manufacturing cells, virtual reality software, Flexible Manufacturing Systems (FMS), computer-aided process planning (CAPP), group technology (GT), Point of Sales (POS) data collection, bar codes, automatic data collection, real time communication/execution systems, Design for Manufacture/Assembly (DFM/A), strategic alliance/based on core/complementary competencies, virtual firm/organization, rapid partnership formation, integration of functions from purchasing to sales, global supply chain management, customer integrated processes for designing, manufacturing, marketing, and support, strategic relationship with customers and suppliers, internal and external cooperation, Business Process Reengineering (BPR), formation of cross function product development teams, concurrent design of products and processes, multidisciplinary team working environment, intelligent engineering design support system, customer and supplier integrated multidiscipline teams, global access to databases and information, Knowledge Based Systems (KBS), knowledge management systems, sensitive information protection, organization structure that promotes innovation, training and education, learning organization, knowledge acquisition from internal and external resources, and core competence management.

**Table 2:** Papers reporting agile manufacturing research on different manufacturing companies.

Serial number	Industry in which agile manufacturing was implemented	Papers reporting the implementation oriented research in agile manufacturing domain	Candidate product	Research reported in the paper
1	Electronic industry	Vinodh et al. [33–45], Deif and ElMaraghy [46], Hooper et al. [47], Khoo and Loi [48], Mondragon et al. [49], Sharifi and Zhang [50], Zandi and Tavana [51]	Several products	Several technologies and models have been applied to achieve agility

2	Pump industry	Vinodh et al. [20], Devadasan et al. [22]	Pump products	Research to infuse agility through the integration of CAD and RPT

3	Aerospace industry	Gunasekaran et al. [52]	Aircraft components	Developed and used an agility audit questionnaire for assessing the agility level of the company
		Mondragon et al. [49]		Studied the impact of information systems in achieving agility

		Sreenivasa et al. [53]		Developed a 30-criterion agility assessment tool
4	Air dryer industry	Sreenivasa et al. [54]	Regenerative air dryer	Reported a research on theoretically mapping air dryer capabilities from agile manufacturing perspectives
		Sreenivasa et al. [55]		Reported a research to enhance the total agility level (TAL) in the company

		Mondragon et al. [49]	Engine and transmission parts	Studied the impact of information systems in achieving agility
5	Automotive industry	Frayret et al. [56]	Motor coach	Demonstrated the effectiveness of a method called “NetMan approach” in acquiring agility characteristics in organisations
		Vinodh and Kuttalingam [9]	Sprocket	Investigated the application of CAD and Computer Aided Engineering (CAE) as enablers of agile manufacturing

6	Machine tool industry	Wang et al. [57]	—	Proposed a multiagent and distributed ruler based approach to carry out production scheduling in agile manufacturing system
		Cheng et al. [58] Cheng et al. [59], Pan et al. [60]	Journal bearings Rolling bearings	Proposed an architecture for implementing agile manufacturing principles by integrating Artificial Intelligence (AI) and internet technology

7	Chemical industry	Guisinger and Ghorashi [61]	—	Described the increasing application of agile manufacturing practices in the specialty chemical industry

8	Casting industry	Yang and Li [62]	—	Researched on evaluating the agility of a company by using the multigrade fuzzy assessment method

9	Household products manufacturing industry	Sharifi and Zhang [50]	Domestic cookers	Designed a conceptual model of agility to enhance the agility of the company

10	Fabrication industry	Fung et al. [63]	Steel frames	Proposed an infrastructure for exercising adaptive production control in an agile manufacturing environment

**Table 3:** Papers reporting agile manufacturing research in electronics manufacturing industry.

Serial number	Papers reporting the research in electronic switch industry	Candidate product chosen for research	Research reported in the paper
1	Vinodh et al. [33], Vinodh et al. [34]	Electronic switch	Designed a model named agile innovative total quality function deployment (agile ITQFD)
2	Vinodh et al. [35]		Designed a tool for quantifying agility in organizations and test its practical compatibility
3	Vinodh et al. [26]		Developed a decision support system named decision support system for quantifying agile criteria (DESSAC)
4	Vinodh et al. [36]		Proposed Computer-Aided Design of Experiment (CADOE) to enable the companies to implement agile manufacturing
5	Vinodh et al. [37]		Research on integrating CAD and CAM to achieve agility in Salzer
6	Vinodh et al. [37]		Research on exploring the interfacing of CAD and CAM to infuse agility in organization
7	Vinodh et al. [38]		Proposed a model named as total agile design system activity based costing model (TADS-ABC)
9	Vinodh [39]		Integrated agility and sustainability to gain business benefits
10	Vinodh et al. [23]		A new concept agile customization program by combining mass customization and agile manufacturing principles
11	Vinodh et al. [40]		Designed and implemented total agile design system (TADS) model
12	Vinodh et al. [41]		Integrated fuzzy logic and analytic hierarchy to identify a group of concepts
13	Vinodh et al. [42]		Assessed agility of an organization using multigrade fuzzy approach
14	Vinodh et al. [43]		Research on achieving agility by carrying out finite element mold analysis
15	Vinodh et al. [44]		Research on examining the possibilities of acquiring design agility characteristics through the aid of CAD
16	Vinodh et al. [45]		Research on using information technology as an enabler of implementing agile manufacturing
17	Deif and ElMaraghy [46]		Developed a system called agile manufacturing planning and control (agile MPC) system

18	Hooper et al. [47]		Explored the operational issues of agile costing systems by conducting a case study in a PCB manufacturing company

19	Khoo and Loi [48]	Computers	Examined the way of enhancing agility in the manufacturing computers using “product modularity” approach

20	Mondragon et al. [49]		Studied the impact of information systems in achieving agility

21	Sharifi and Zhang [50]	Electronic components	Designed a conceptual model of agility which consisted of three elements, namely, agility drivers, agility capabilities, and agility providers

22	Zandi and Tavana [51]	Semiconductors	A research on evaluating the best electronic-customer relationship management (e-CRM) network for achieving agility
